# Taxonomy and Polyphasic Characterization of Alkaline Amylase Producing Marine Actinomycete *Streptomyces rochei* BTSS 1001

**DOI:** 10.1155/2013/276921

**Published:** 2013-12-30

**Authors:** Aparna Acharyabhatta, Siva Kumar Kandula, Ramana Terli

**Affiliations:** ^1^Department of Biotechnology, Dr. L. Bullayya College, New Resapuvanipalem, Visakhapatnam, Andhra Pradesh 530013, India; ^2^Department of Biotechnology, Andhra University, Visakhapatnam 530003, India; ^3^School of Life Sciences, GITAM University, Visakhapatnam 530045, India

## Abstract

Actinomycetes isolated from marine sediments along the southeast coast of Bay of Bengal were investigated for amylolytic activity. Marine actinomycete BTSS 1001 producing an alkaline amylase was identified from marine sediment of Diviseema coast, Bay of Bengal. The isolate produced alkaline amylase with maximum amylolytic activity at pH 9.5 at 50°C. The organism produced white to pale grey substrate mycelium and grayish aerial mycelium with pinkish brown pigmentation. A comprehensive study of morphological, physiological parameters, cultural characteristics, and biochemical studies was performed. The presence of iso-C_15 : 0_, anteiso-C_15 : 0_, iso-C_16 : 0_, and anteiso-C_17 : 0_ as the major cellular fatty acids, LL-diaminopimelic acid as the characteristic cell wall component, and menaquinones MK-9H_(6)_ and MK-9H_(8)_ as the major isoprenoid quinones is attributed to the strain BTSS 1001 belonging to the genus *Streptomyces*. Comparison of 16S rRNA gene sequences showed that strain BTSS 1001 exhibited the highest similarities to the type strains of *Streptomyces rochei* (99%), *Streptomyces plicatus* (99%), and *Streptomyces enissocaesilis* (99%). Using the polyphasic taxonomical approach and phenotypic characteristic studies, the isolate BTSS 1001 was characterized as marine actinomycete *Streptomyces rochei*.

## 1. Introduction

Actinomycetes have long been reported as important source of biotechnologically important compounds. The recent focus is on marine Actinomycetes as a source of bioactive compounds and industrial enzymes. This is due to the fact that terrestrial actinomycetes have been exhaustively analyzed for bioactive compounds and enzymes but they still fall short of industrial application. Thus, the need of the hour is to identify newer sources capable of withstanding the conditions of industrial and commercial applications. Studies by several researchers [1–4] on marine actinomycetes have reported diversity and presence of unique marine taxa in ocean sediments. Their survival in extreme conditions in the ocean sediments demonstrates their ability for adaptation and production of different types of bioactive compounds as compared to their terrestrial counterparts [5]. Marine actinomycetes have been established as a rich source of several secondary metabolites such as novel bioactive molecules like antibiotics, antifungal, and anticancer compounds, plant growth hormones, industrially important enzymes, enzyme inhibitors, and pigments [6, 7]. Culturally independent methods and polyphasic approaches have also demonstrated that marine sediments contain wide range of unique microorganisms [5, 8, 9] producing natural metabolites. The polyphasic approach helps determine the taxonomy of the species within the genus [10].

*α*-Amylases (1,4-*α*-D-glucan glucanohydrolases, E.C. 3.2.1.1) are one of the most important industrial enzymes. They cleave internal *α*-1,4-glycosidic linkage in starch. Their application potential and market value in various industries have been widely explored [11, 12]. Alkaline *α*-amylases have high catalytic efficiency and stability at the alkaline pH ranging from 9.0 to 11.0 [13] and hydrolyze starch under high pH conditions in the starch and textile industries and also as ingredients in detergents for automatic dishwashers and laundries [14, 15]. Due to low yield and stability of alkaline amylases from wild type strains, recombinant technology has been actively employed to increase yield of alkaline amylase production [15]. While indigenous alkaline *α*-amylases have mostly been reported from a wide range of bacterial strains [16–22], very few studies on Streptomyces have been reported. Most amylases reported from *Streptomyces* sp. are active in the pH ranges of 5.0–7.5, with limitations for industrial applications. So far, only limited studies have been reported on alkaline amylase producing *Streptomyces *[22–26]. The marine sediments of Bay of Bengal have proved to be a productive source [27] in isolation of potential species with novel bioactive properties and industrial applications. The current studies mainly focus on tapping marine sediments as a source for isolating and characterizing actinomycete strains capable of producing alkaline amylase suitable for industrial production. The screening and characterization studies resulted in an alkaliphilic and moderately thermostable marine actinomycete BTSS 1001 strain. The strain shares similarity with *Streptomyces rochei* and produces thermostable alkaline amylase. The strain was characterized in a polyphasic taxonomy to study its uniqueness and suitability as a potential source of industrial enzymes.

## 2. Materials and Methods

### 2.1. Collection of Marine Samples

The marine sediments were collected along the southeast coast of Bengal Bay, India, at various depths ranging from 50 m to 200 m using a grab sampler. The sediments were stored in sterile zipped plastic bags. The soil sediments were subjected to heat pretreatment at 50°C for 60 min for isolation of marine actinomycetes. One gram of each soil samples was then suspended in sterile water and incubated at 28°C in a rotary shaker at 150 rpm for 1 hour. The suspension was serially diluted up to 10^−7^ level. 0.1 mL of each of these dilutions was plated on selective media such as actinomycetes isolation medium, glycerol yeast extract agar, starch casein agar, and glucose asparagine agar. All the media were prepared using 50% (v/v) aged, filtered (0.20 *μ*m) sterilized sea water; the isolation media plates were also supplemented with rifampicin 5 *μ*g/mL and nystatin 25 *μ*g/mL to inhibit bacterial and fungal contamination, respectively. The pH of the media was maintained at 7.9. The petri plates were incubated at 28°C and are observed from one week to three weeks for characteristic colonies of Actinobacteria.

The isolated marine actinomycetes were maintained on starch casein agar and yeast extract malt extract agar slants over laid with 10% glycerol. A total of 10 marine isolates were selected for further study of amylolytic activity. The sampling locations of the isolates are given in Table 1.

### 2.2. Screening for Amylolytic Marine Actinomycetes

The strains were tested for their ability to degrade amylase by starch agar plate method supplemented with 1% starch and submerged culture studies. The production media for submerged culture consisted of (g/L) 10.0 soluble starch, 2.0 yeast extract, 0.5 MgSO_4_, 0.5 MnSO_4_, 1.0 KH_2_PO_4_, 30 NaCl, 0.02 FeSO_4_, and 0.1 CaCl_2_ with a pH of 7.9. The flasks were inoculated with 2% (v/v) spore suspensions of 2.0 × 10^6^ spores/mL and incubated at 28°C in a rotary shaker at 200 rpm for 96 hrs.

### 2.3. Amylase Activity

The amylase activity was evaluated by determining the amount of reducing sugar released from starch hydrolysis [29]. The culture was centrifuged at 8,000 rpm for 10 min at 4°C. The resultant cell free supernatant was used as crude enzyme. Amylase activity was assayed by adding 0.5 mL crude enzyme to 0.5 mL of 1% starch in 50 mM Glycine-NaOH buffer pH 9.5 and incubated at 60°C for 15 min. The reaction was stopped by addition of 1 mL DNS (3,5-dinitrosalicylic acid). The activity was determined by estimating the liberation of reducing sugars as a result of amylase action on starch and expressed in units. (One unit of alpha amylase activity was defined as the amount of enzyme that releases 1 *μ*g of maltose per mL per min under the assay conditions).

### 2.4. Estimation of Extra Cellular Protein

Concentration of protein was estimated by the method of [30] and by using standard BSA solution to measure protein concentration.

### 2.5. Polyphasic Characterization of the Active Isolate BTSS 1001

#### 2.5.1. Morphological, Cultural, Biochemical, and Physiological

The active strain BTSS 1001 was characterized up to genus level by observing the color of the aerial spore mass, diffusible pigments, and substrate mycelia pigmentation as described by [31] and International Streptomyces Project (ISP). The morphological characteristics of strain BTSS 1001, including spore-chain morphology, spore size, and surface ornamentation, were assessed by scanning electron microscopy (model JSM-6610 LV; JEOL, Ltd., USA) of 14- and 28-day-old cultures prepared on ISP 2 medium. A range of physiological and biochemical characteristics were examined according to the standard protocols of [32–34].

#### 2.5.2. Analysis of Chemotaxonomic Characteristics

The Culture was grown on TSB agar and cells were analyzed for chemotaxonomic characteristics. Cell wall amino acids and sugars were isolated to analyze the isomeric forms of diaminopimelic acid [35] and whole-organism sugars [36]. The major membrane-associated menaquinones were determined by established protocols [37, 38]. Fatty acids were extracted, methylated, and analysed by GC using the standard methods [39, 40] and the results were compared with the fatty acid database of the microbial identification system.

### 2.6. Genomic DNA Isolation

The protocol for DNA isolation was followed according to methods devised by [9] with slight modifications. The genomic DNA pellet was dissolved in 200 *μ*L of Tris-EDTA for further analysis and storage at −20°C.

### 2.7. Phylogenetic Analysis Based on 16S rRNA Sequences

PCR amplification and sequencing of the 16S rRNA gene were performed as described by Li et al. [41]. The partial 16S rRNA gene sequence (1,529 nucleotides) was used to search the GenBank database with the BLAST algorithm [42] to determine relative phylogenetic positions. Multiple alignments with sequences of the most closely related Actinobacteria and sequence similarity calculations were carried out using CLUSTAL X [43]. The phylogenetic trees were constructed by the neighbor-joining and maximum-likelihood [44] tree-making algorithms using the software package PHYLIP version 3.69 [45] and viewed in Treeview [46]. The topologies of the phylogenetic trees were evaluated using the bootstrap resampling method of Felsenstein [47] with 1000 replicates.

#### 2.7.1. GenBank Submission

The 16S rRNA sequence was submitted in GenBank with the accession number: JX284411.

## 3. Results

### 3.1. Screening for Amylolytic Marine Actinomycetes

**Figure 1:**
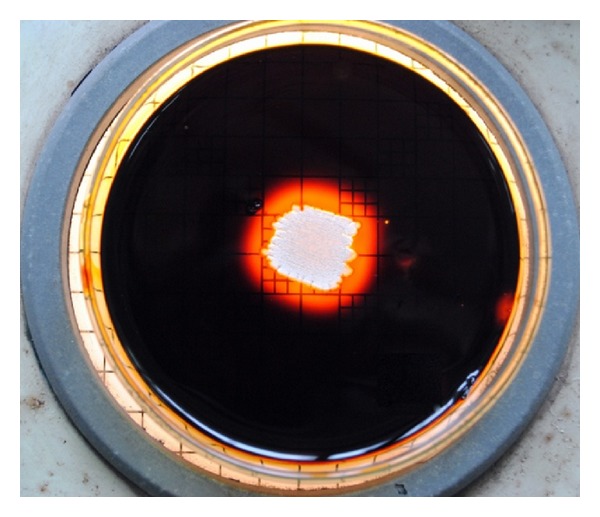
Starch agar plate showing amylase activity with clear zone at the center surrounding the culture of BTSS 1001 isolated from marine sediment of Bay of Bengal.

**Figure 2:**
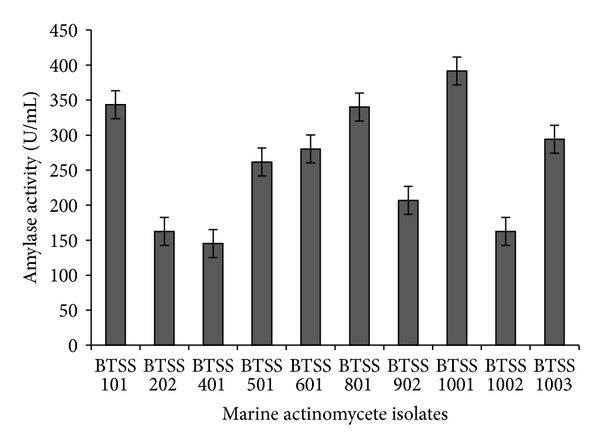
Alkaline amylolytic activity of marine actinomycete isolates under submerged fermentation.

Among the samples screened strains, BTSS 1001 is showing the highest activity on starch agar plates (Figure 1). Further secondary screening confirmed the production of amylase and the results are given in Figure 2. The results show that the isolate BTSS 1001 exhibited maximum enzymatic activity (391.45 U/mL) followed by BTSS 101 (343.59 U/mL) and BTSS 801 (340.17 U/mL) under the same conditions.

### 3.2. General Characteristics of Active Isolate BTSS1001

**Figure 3:**
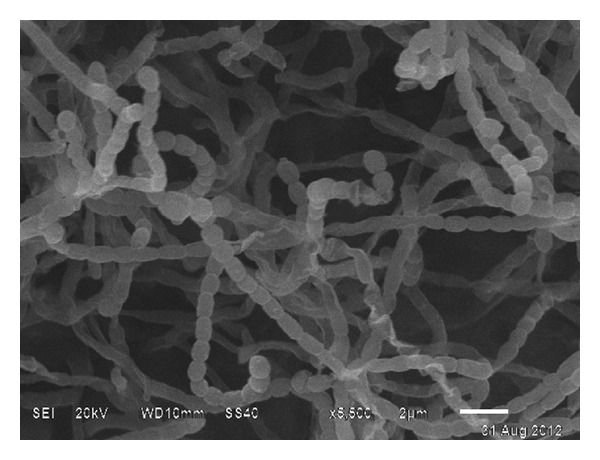
Scanning electron micrograph of BTSS 1001, a marine actinomycete grown on ISP 2 media for 3 weeks.

Aerobic, nonmotile, Gram-positive actinomycete forms extensively branched substrate and aerial mycelia. The aerial mycelium carries smooth spiral spores. The average diameter of the spores is around 0.9 mm. The spore chain consists of 10–12 spores/chain (Figure 3). Strain BTSS grew well on media ISP 2, ISP 3, ISP 4, and ISP 5 [30], but not on ISP 1 and nutrient agar medium at 32°C as the media constituents were unable to support the growth of the organism as they were lacking the required mineral salts. Lavender to reddish brown diffusible pigments were observed ISP 2, ISP 7, and starch casein agar on incubation period of one month. Isolate BTSS1001 formed white to pale grey substrate mycelia and mouse grey aerial mycelium with lavender pigmentation on starch casein agar plates which is characteristic of *Streptomyces* sp. The growth pattern and cultural characteristics on different ISP media are given in Table 2.

**Figure 4:**
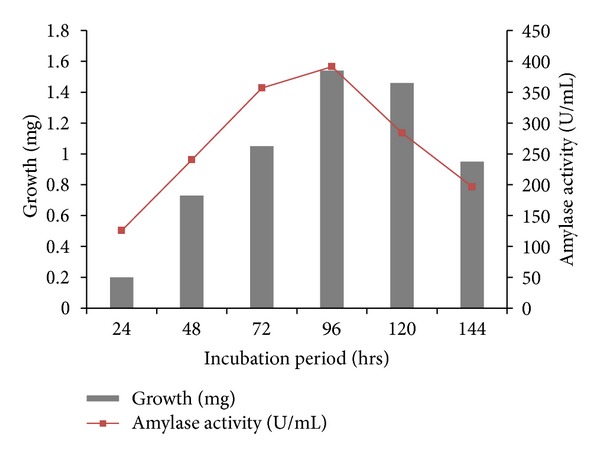
Growth and Amylase activity of BTSS 1001 at various incubation periods.

**Figure 5:**
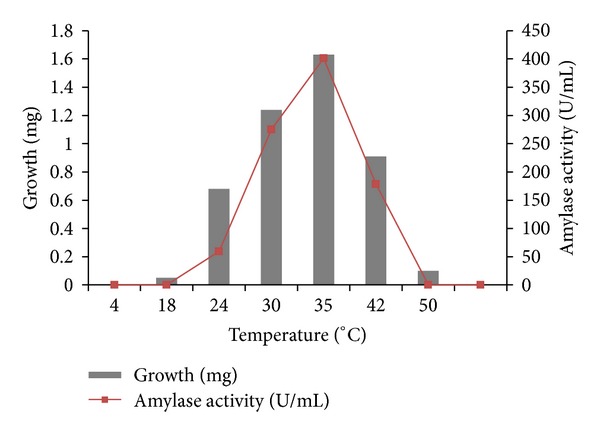
Effect of incubation temperature on growth of BTSS1001 and amylase production.

**Figure 6:**
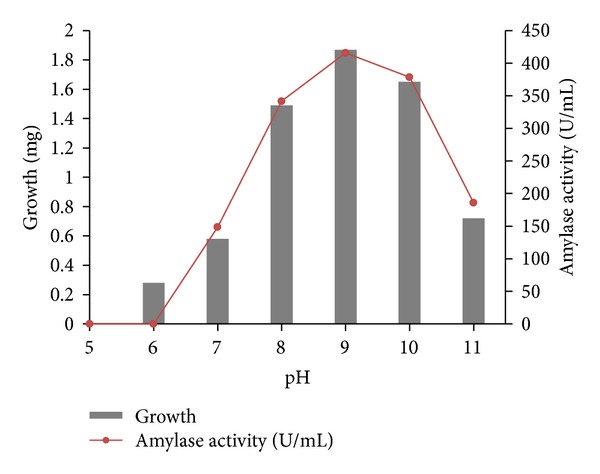
Effect of initial pH on growth and amylase activity of BTSS 1001.

**Figure 7:**
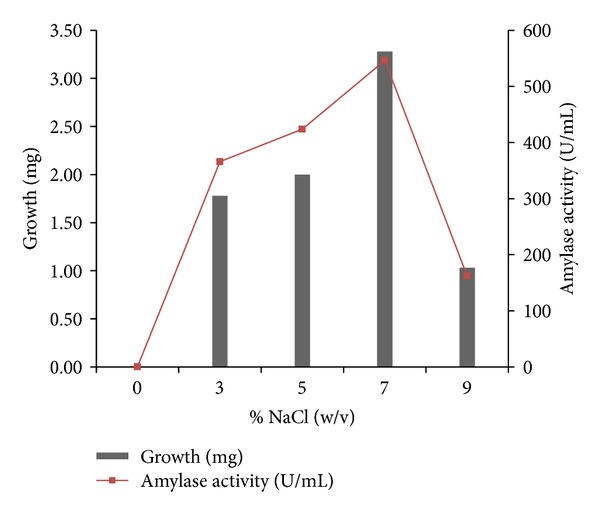
Effect of NaCl concentration on growth and amylase activity.

Detailed physiological and biochemical properties of the strain are given in the species description (Table 3). The isolate was able to utilize most carbon sources except for rhamnose. Acid production from sugars was positive only with glucose and xylose. It was unable to convert all other sugars. The isolate BTSS 1001 shows cultural similarity with *Streptomyces rochei* but exhibits differences in biochemical and physiological properties. The isolate showed optimum growth on 96 hrs incubation period (Figure 4), at a temperature range of 25–42°C (Figure 5) (optimum 35°C), at pH 8.0–10.5 (Figure 6) (optimum pH 9.0), and with 3–10% (w/v) NaCl (Figure 7) (optimum 7% w/v).

### 3.3. Chemotaxonomic Studies

The cell wall amino acids, sugars, menaquinones, and fatty acid components of the strain were analyzed. The amino acids of the cell wall were LL-diaminopimelic acid. No characteristic whole cell sugars were detected. Analysis of menaquinones and fatty acids showed the predominant menaquinones (isoprenoid quinones) of BTSS 1001 strain as MK-9(H_6_) and MK-9(H_8_). The fatty acid profile showed presence of iso-branched, anteiso-branched, and saturated fatty acids. The major cellular fatty acids were found to be iso-C (14 : 0)—8.37%; iso-C (15 : 0)—10.12%; anteiso-C (15 : 0)—23.84%; iso-C (16 : 0)—22.28%; C (16 : 0)—6.02%, and anteiso-C (17 : 0)—6.49%. The strain was deposited in Microbial Type Culture Collection and Gene Bank (MTCC), India, as MTCC 36855.

### 3.4. Molecular Taxonomy of Strain BTSS 1001

**Figure 8:**
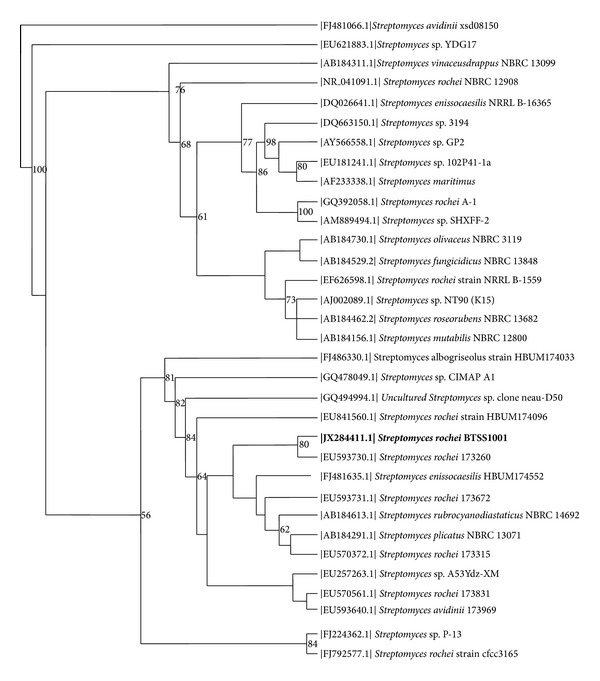
Phylogenetic tree of strain BTSS 1001 and its closest relatives within the family Streptomycetaceae, reconstructed by using the neighbour-joining method, based on 16S rRNA gene sequences. Numbers at nodes are bootstrap percentages based on 1000 resamplings (only values of 50% or more are indicated).

BLAST result of the nearly complete 16S rRNA gene sequences (averaging 1,529 nucleotides) of the strain BTSS 1001 against sequences in the GenBank database revealed homologies of 99% to members of the family Streptomycetaceae. The BLAST result gave similarity matches to *Streptomyces rochei* (99.00%), *Streptomyces plicatus *(99.00%), *Streptomyces olivaceus* (99.00%), and *Streptomyces enissocaesilis* (99.00%). Multiple alignment of the highly similar sequences in clustal X and maximum parsimony studies generated bootstrap values which were used to construct the phylogenetic tree. The phylogenetic tree generated showed the closest neighbor branching to *Streptomyces rochei* 173260, (GeneBank accession no. EU593730.1), isolated from Xinjiang, China, with 80% bootstrap value. The strain also showed branching with 64% bootstrap value with *Streptomyces enissocaesilis* HUMB 174552, *Streptomyces plicatus* NBRC 13071, and other reported strains of *Streptomyces rochei*. The phylogenetic tree (Figure 8) of BTSS 1001 16S rRNA sequence suggested that the isolate is placed under the genus *Streptomyces* and is placed closest to *Streptomyces rochei*.

## 4. Disscussion

The diversity of marine actinomycetes is significant in several areas of science and medicine [9]. They are a rich source for chemically diverse bioactive compounds [48]. Studies on marine sediments revealed *Streptomyces* as potential producers of amylolytic enzymes. The marine isolate produced amylase enzyme which had maximum activity at pH 9.5 and temperature of 50°C. The activity is in comparable levels to thermostable, alkaline bacterial amylases and lends the isolate *Streptomyces rochei *as a potential microbe to produce alkaline amylase required for high temperature and pH applications. The functionality of the enzyme at elevated temperatures improves the solubility of starch, decreases the viscosity, limits microbial contaminants, and reduces reaction time [49].

A polyphasic approach in characterizing the isolate enabled identifying the organism up to species level. The cultural characteristics of pigmentation, colony morphology, and spore morphology placed the strain in the family Streptomycetaceae. The cell wall sugars, menaquinones, and fatty acid profile placed the organism under genus *Streptomyces. *The organism showed similarity in morphology to *Streptomyces rochei* (Berger et al. 1953) [28]. The complete physiochemical and biochemical properties and analysis of 16S rRNA gene sequences supported this. Furthermore, 16S rRNA gene analysis and phylogenetic studies showed homology to three different species of *Streptomyces rochei, Streptomyces plicatus*, and *Streptomyces enissocaesilis.* Based on the polyphasic approach, the strain is identified as *Streptomyces rochei.* The significance of the study is that the isolate showed varied requirements as compared to *Streptomyces rochei* (Berger et al. 1953) [28] and other reported strains. The marine strain required mesophilic conditions of 37°C for growth in accordance with (Williams et al.) [34] that actinomycetes behave as mesophiles. An alkaline condition of 8.0–10.0 was suitable for growth and enzyme production. The preference of alkaline environment for growth and enzyme production may be attributed to the marine environment at source and hence the divergence from terrestrial counterparts. The moderately halophilic marine organisms grow over a NaCl concentration from 5 to 20%. The isolate BTSS 1001 showed salt tolerance up to 9%, with an optimum at 7%, and so it may be placed in the moderate salt tolerant halophilic group and salt tolerance indicates the strain as an indigenous marine species. The organism was characterized as an alkaliphilic, moderately salt tolerant actinomycete *Streptomyces rochei*. A major goal of examining the marine actinomycetes was due to the fact that the microbial diversity of marine species also shows chemical diversity. We establish from this study that marine species show diversity from terrestrial counterparts and that the metabolites will also show diversity in structure and function. This is the first study reported so far on alkaline amylase production by *Streptomyces rochei* isolated from marine sediment. The activity levels and alkaline nature of the enzyme show promising scope in various industrial applications. The characterization of the isolate was done to establish the indigenous nature of the marine isolate and scope of marine sediments as a rich source of amylolytic actinomycetes. Further characterization studies on the alkaline amylase are being conducted for media optimization studies, evaluation of enzyme novelty, and protein structure elucidation.

## Figures and Tables

**Table 1:** Sampling locations.

Isolate	Location	Depth (meters)	Latitude	Longitude

BTSS 101	1	265	17°50.814 N	84°01.422 E
BTSS 202	2	52.93	17°50.556 N	83°01.228 E
BTSS 401	4	201.17	16°59.832 N	82°58.065 E
BTSS 501	5	108.05	16°59.507 N	82°43.923 E
BTSS 601	6	50.87	16°59.805 N	82°32.497 E
BTSS 801	8	191	15°59.813 N	81°29.045 E
BTSS 901	9	88.11	15°59.813 N	81°24.737 E
BTSS 1001	10	62.72	15°59.481 N	81°22.716 E
BTSS 1002	10	62.72	15°59.481 N	81°22.716 E
BTSS 1003	10	62.72	15°59.481 N	81°22.716 E

**Table 2:** Morphology of marine isolate BTSS 1001 on various ISP media.

S. no.	ISP media	Aerial mycelium	Substrate mycelium	Pigmentation	Spore formation	Growth
1	Tryptone yeast extract agar (ISP 1)	Nil	Grey	Nil	Poor	Poor
2	Yeast extract malt extract agar (ISP 2)	Lavender to grey	Light grey	Reddish brown (28 days)	Good	Good
3	Oat meal agar (ISP-3)	Dark grey	Grey	Brown	Good	Good
4	Inorganic salt starch agar (ISP-4)	Light grey	White	Nil	Moderate	Good
5	Glycerol asparagine agar (ISP 5)	Grey	White	Nil	Good	Good
6	Tyrosine agar (ISP-7)	Grey	White	Brown (28 days)	Good	Good
7	Starch casein agar	Grey	Lavender grey	Lavender brown (15 days)	Good	Good

**Table 3:** Morphological, biochemical, and physiological characteristics of marine isolate BTSS 1001 in comparison with *Streptomyces rochei* (Berger et al., 1953 [28]).

Properties	BTSS 1001	*Streptomyces rochei* (Berger et al., 1953 [28])
Spore chain	Spiral	Spiral
Spore mass	Grey	Grey
Substrate mycelium	Light grey	Light grey
Ariel mycelium	Dark grey	Dark grey
Soluble pigment	Lavender to brown	Nil
Growth at temperature	25°C–42°C optimum 35°C	Optimum 28°C
Growth at pH	8–11	6-7
NaCl conc.	Up to 7%	Up to 4%
Biochemical characteristics
Indole production	−	−
Methyl red	−	−
Voges proskauer	−	−
Citrate utilization	+	+
H_2_S production	−	−
Nitrate reduction	−	−
Urease	+	+
Catalase	+	+
Oxidase	+	+
Melanin production	+	−
Starch hydrolysis	+	+
Gelatin hydrolysis	+	+
Lipid hydrolysis	+	+
Casein hydrolysis	−	−
Haemolysis	+	+
Carbon source utilization
Arabinose	++	++
Fructose	+ +	+ ++
Glucose	+++	++ +
Galactose	+	+++
Glycerol	++++	++++
Lactose	+++	+++
Maltose	+++	+++
Mannitol	++	+++
Ribose	+	+
Raffinose	++	++
Rhamnose	−	−
Starch	++++	+++
Acid production
Glucose	+	+
Fructose	−	+
Mesoinositol	−	+
Mannitol	−	+
Rhamnose	−	+
Xylose	+	+
